# What Is the Impact of Leaders with Emotional Intelligence on Proxy Performance Metrics in 21st Century Healthcare?—A Systematic Literature Review

**DOI:** 10.3390/ijerph21111531

**Published:** 2024-11-18

**Authors:** Aisha Chaudry, Parisah Maham Hussain, Simran Halari, Sohini Thakor, Aran Sivapalan, Abdul Ikar, Terrell Okhiria, Edgar Meyer

**Affiliations:** 1 Hull University Teaching Hospitals NHS Trust, Kingston upon Hull HU3 2JZ, UK; 2 Royal Free London NHS Foundation Trust, London NW3 2QG, UK; parisah_maham@hotmail.co.uk; 3 Chelsea and Westminster Hospital NHS Foundation Trust, London SW10 9NH, UK; 4 Sandwell and West Birmingham Hospitals NHS Trust, Birmingham B71 4HJ, UK; 5 Lewisham and Greenwich NHS Trust, London SE13 6LH, UK; 6 NHS North East London Clinical Commissioning Group, London N1 5LZ, UK; 7 Queen Elizabeth Hospital King’s Lynn NHS Foundation Trust, King’s Lynn PE30 4ET, UK; trokhiria@gmail.com; 8 Birmingham Business School, University of Birmingham, Birmingham B15 2TT, UK

**Keywords:** emotional intelligence (EQ), leadership, healthcare management, employee commitment, burnout, organisational dynamics, team empowerment, healthcare outcomes, performance improvement, systematic review, workforce wellbeing, patient outcomes, leadership development, healthcare environment

## Abstract

Emotional intelligence (EQ) in healthcare leadership has been a subject of debate regarding its significance in enhancing job performance and patient-centred care. This systematic review investigates the impact of EQ on organisational performance metrics in healthcare leaders. Eleven studies meeting the inclusion criteria were identified through a comprehensive database search. The findings suggest that EQ positively influences job satisfaction, with emotionally intelligent leaders fostering a positive work environment and commitment among employees. Moreover, EQ correlates negatively with emotional exhaustion, indicating its potential in mitigating burnout rates among healthcare professionals. EQ fosters teamwork, organisational culture and enhances job performance, with higher EQ levels in leaders associated with increased team empowerment and proactivity. Despite the compelling evidence, limitations in the study methodologies and heterogeneity in the reported outcomes challenge the establishment of definitive conclusions. Nevertheless, the findings underscore the importance of EQ in healthcare leadership and its potential to improve organisational dynamics and employee wellbeing. This review highlights the need for further research on EQ’s impact on patient satisfaction and calls for the development of EQ training programmes tailored for healthcare leaders.

## 1. Introduction

The controversy surrounding emotional intelligence’s (EQ) potential role in healthcare is contentious, with proponents and opponents debating its significance. In the 21st Century, arguably non-clinical skills, such as leadership and EQ, are crucial for healthcare professionals as they enhance job performance and patient-centred care [1]. The necessity for leadership in healthcare is omnipresent, as evidenced by the positive impact of enhanced leadership on patient safety outcomes and the overall quality of care [2], with many healthcare leaders and stakeholders supporting the adoption of EQ due to its diverse range of non-clinical competencies [3,4]. Also, EQ has been established as a significant element contributing to the achievement of successful leadership. High levels of EQ are frequently found among individuals who exhibit a transformative style of leadership [5]. Additionally, EQ is considered a critical aptitude for healthcare professionals, irrespective of their engagement in either formal or informal leadership roles [6]. As direct measurement of EQ’s influence on metrics such as the mortality rate is not feasible, this systematic literature review (SLR) primarily investigates proxy performance metrics to explore any potential importance of EQ.

The findings of this SLR aim to contribute valuable evidence to the relatively young field of research on EQ in healthcare. By examining four proxy performance metrics, namely, job satisfaction, emotional exhaustion, organisational culture and job performance, the study seeks to develop the evidence base on the relevance of leaders’ EQ in the healthcare context.

## 2. Materials and Methods

The present study is a systematic literature review that has been registered with PROSPERO, identified by CRD Number CRD42023416455. The researchers searched five electronic databases: Medline, EMBASE, HMIC, Scopus and Web Of Science. While EMBASE, HMIC and Web Of Science primarily cover journals in the fields of biomedical science, the social sciences and management, Medline and Scopus are focused on the discipline of health sciences. Database email alerts were set up to keep track of newly published articles. Screening of the references and citations in all the included papers was undertaken to ensure that any pertinent research that was not identified through database searching was not missed. 

Keywords were identified using the PICO framework, and search strings were constructed accordingly for each keyword [Appendix A]. The researchers used Boolean operators to combine the search terms. The study considered English papers published over a 15-year period spanning 2009 to 2023.

To systematically manage the retrieved papers, EndNote was utilised. During the screening process, inclusion criteria were applied based on the title and abstract of the papers (Table 1). Papers meeting the initial criteria were subjected to further evaluation through full-text assessment using the same inclusion and exclusion criteria. This phase of the review involved three to four researchers who independently assessed and discussed the papers, employing a collaborative approach to minimise errors in inclusion and exclusion decisions. Any disagreements that arose were resolved through discussion.

The critical appraisal of each paper was conducted independently by two researchers within the group, using the JBI checklist [7] to reduce subjectivity. In cases of uncertainty between the two researchers, a third independent researcher was consulted to reach a consensus.

Ideally, meta-analyses would have been performed as they are considered the pinnacle of evidence hierarchy, providing more robust and convincing conclusions. However, due to the lack of homogeneity in the results, an inductive thematic approach was adopted to discuss the findings.

## 3. Results

**Figure 1:**
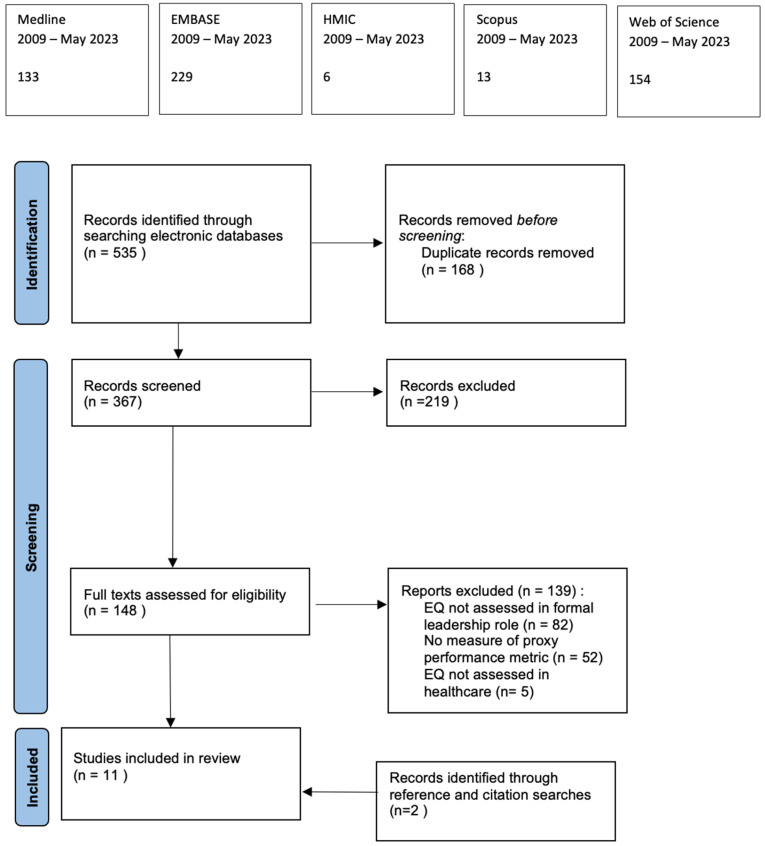
PRISMA flow diagram for systematic review.

The search retrieved 535 records, of which 368 abstracts were evaluated by the author with regard to their compatibility with the inclusion criteria (Table 1). Subsequently, 148 full texts underwent assessment for eligibility. Following screening of the reference lists and citations, a total of 11 studies were found to assess the impact of leaders’ EQ on proxy performance metrics (Figure 1). Ten of these studies collected data at a single point in time using structured questionnaires. The remaining paper was a prospective longitudinal cohort study (Table 2).

### Quality Assessment 

The JBI findings for each study are detailed in Table 3. Among the eleven studies incorporated, bias was evident in ten of them. The presence of heterogeneity and the diverse reporting standards across the studies constrained the extent to which meaningful comparisons could be made.

## 4. Discussion

### 4.1. The Influence of a Healthcare Leader’s Emotional Intelligence on Satisfaction

**Figure 2:**
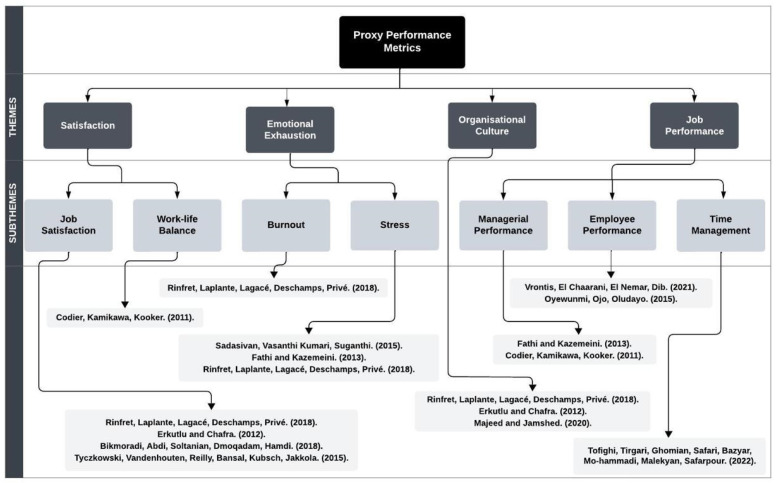
Thematic analysis flow diagram of proxy performance metrics [8,9,10,11,12,13,14,15,16,17,18].

The impact of EQ in healthcare leaders on a leader’s satisfaction was examined in a collection of five papers, comprising four cross-sectional studies and one prospective longitudinal study [10,11,12,13,18]. As depicted in Figure 2, four papers investigated the influence of EQ on job satisfaction, whilst one study focused on satisfaction related to work–life balance. All four papers focusing on job satisfaction consistently found that higher levels of EQ contribute to increased job satisfaction for both leaders and their employees.

A large cross-sectional study, involving 370 nursing managers, concluded that EQ competencies enable nurse managers to approach daily tasks and situations with a positive attitude, resulting in greater satisfaction with their work [13]. Another study involving 171 executive healthcare directors demonstrated a causal relationship, showing how EQ indirectly influences job satisfaction [10]. The study found that high EQ in executive directors was associated with the display of transformational leadership (TL) qualities. Consequently, transformational leadership was linked to greater perceived organisational justice, leading to greater job satisfaction, work commitment and self-determined motivation. Thus, Rinfret et al. [10] proposed that EQ plays an upstream role in this presumed causal pathway. A further cross-sectional study demonstrated similar findings with regard to the relationship between EQ and transformational leadership. The study, involving 77 nurse managers in the USA, revealed that EQ accounted for 44% of the variation in transformational leadership style and indicated a positive association between the self-expression sub-domain of EQ and both transformational leadership style and leadership outcomes, specifically, “extra effort”, “effectiveness” and “satisfaction” (measured using the “Outcomes Of Leadership’’ scale) [18]. Building on this conclusion, emotionally intelligent leaders possess the skill to inspire and motivate team members, especially those who may have felt excluded in the past. Such leaders cultivate a culture of resilience and creativity, fostering a sense of pride within the team. The positive support offered by these leaders significantly contributes to increased job satisfaction among employees [12].

The findings in our review align with those of Louwen et al. [19], who highlight the importance of personality traits, behaviour styles and emotional intelligence in shaping professional dynamics within healthcare. Their systematic review underscores how EQ, combined with behaviour and personality profiling, can foster a supportive and positive work environment, enhancing job satisfaction and team cohesion. Integrating these insights with our findings, it is clear that EQ in healthcare leaders plays a crucial role in facilitating job satisfaction, a critical component of effective leadership in healthcare settings.

However, given the cross-sectional nature of most studies, causal evidence remains tentative. A prospective longitudinal study examining EQ and peer coaching also supports a positive correlation between EQ and work–life balance satisfaction [11]. Synthesising these outcomes suggests EQ enhances both work and life satisfaction.

### 4.2. The Influence of a Healthcare Leader’s Emotional Intelligence on Emotional Exhaustion 

One paper assessed the correlation between EQ in leaders and burnout. In a cross-sectional study conducted in Quebec, Rinfret et al. [10] discovered a significant correlation between the EQ of executive directors (EDs) and reduced burnout rates among both individual employees and those in their proximity. The study highlighted that higher EQ in leaders was associated with a perceived improvement in TL skills as reported by employees. This perceived increase in TL skills, in turn, leads to a higher perception of organisation changes being implemented fairly. This fosters commitment to change and enhances the intrinsic motivation of employees, which was linked to a decrease in levels of emotional exhaustion, demonstrating the considerable impact of EQ on burnout rates within healthcare teams. The direct correlation between EQ and emotional exhaustion was −0.29 (*p* < 0.01). However, it is important to acknowledge the potential presence of selection bias in the study, as EDs selectively chose which employees were surveyed, raising the possibility that more committed employees may have been included. A prospective longitudinal study conducted at a private tertiary centre in Hawaii identified high scores in burnout-specific variables. Whilst the study may provide some support for the hypothesised relationship between emotional intelligence (EQ) and lower burnout rates, this particular correlation was not analysed in the study [11].

The relationship between emotionally intelligent healthcare leaders and occupational stress was explored within three cross-sectional studies [8,9,10]. All the papers found that a higher level of EQ positively impacts the levels of stress, either through a direct reduction in stress or through tools to manage stress effectively. A cross-sectional study that conducted surveys of managers of the Ministry of Health and Medical Education found a significant and negative correlation between emotional intelligence and stress but also between stress and performance [9]. These were echoed in a similar cross-sectional study which found that EQ was associated with work stress and helped leaders to become conscious of their emotions in order to reduce stress [8]. 

### 4.3. The Influence of a Healthcare Leader’s Emotional Intelligence on Teamwork and Organisational Culture 

Three papers assessed the role of a leader’s EQ on teams, with all the studies showing that higher EQ in healthcare leaders can have positive impacts on teams and organisational culture [10,12,14]. A cross-sectional study with a notably large sample size of 910 participants assessed the moderating role of a leader’s emotional intelligence on team empowerment [12]. The study found that higher EQ in leaders resulted in increased team meaningfulness, which in turn increased team empowerment and team proactivity. The critical study concluded that EQ is an essential skill for those organisations wishing to increase the level of personal initiative and team proactivity in the workplace. A further cross-sectional study supported this conclusion, showing that employees’ perceptions of leaders with a higher EQ were positively related to their perceptions of leaders’ TL and this leads to a higher perception of organisational changes being implemented fairly, in turn having positive effects on teamwork [10]. These findings were echoed in a further cross-sectional study with a large sample size of 344 nurses. The study examined the mediation of team culture in the relationship between leader emotional intelligence and turnover intention, with the aim of determining the significance of the results. The findings provided empirical evidence supporting the proposed mediating role of team culture. The results indicated that leader emotional intelligence has a positive impact in reducing turnover intentions among nursing staff and strengthens this relationship by fostering a conducive team culture [14].

### 4.4. The Influence of a Healthcare Leader’s Emotional Intelligence on Job Performance

The impact of leaders’ EQ on job performance was examined across five papers. Among these studies, four were cross-sectional, and one was a cohort study. The prospective longitudinal cohort study assessed the influence of a peer coaching intervention on the EQ of 15 nurse managers over a period of six months. According to the study by Codier et al., all the participating managers showed improvement in their managerial skills and emotional abilities as a result of the intervention [11]. However, some limitations in the study raise concerns. Firstly, the sample size was small, and the attrition rate exceeded 50%. Moreover, the measurement of emotional abilities in the study remains unclear, as it appears to rely on qualitative perceptions of the nurse managers. Although the participants reported a perceived improvement in their EI abilities, the study later contradicts this claim by stating that their scores actually decreased over the study period. As a result, drawing reliable conclusions from this study becomes problematic. In an additional study investigating the link between EQ and managerial performance, surveys were administered to assess the relationship between EQ and the performance of directors within the Health and Medical Education Ministry [9]. The findings demonstrated a statistically significant and positive correlation between EQ and directors’ performance. EQ was found to have both a direct impact (with a measurement of 0.612) and an indirect impact (through stress reduction) on the directors’ performance. The study boasted a relatively large sample size, and the use of cluster sampling added to the study’s reliability. However, the study’s findings regarding the measurement and nature of performance are inconclusive, making it difficult to draw definitive conclusions in this regard. 

In a cross-sectional study involving 38 head nurses of emergency and intensive care units in Iran, researchers examined the link between EQ levels and time management skills [16]. Interestingly, contrary to studies conducted in non-nursing contexts by Arefi et al. [20], Bahadir [21], Ghodrati and Najafzadeh [22], Godini and Baghfalaki [23], Kavousy et al. [24], Mousavi et al. [25] and Nazem and Ghaed Mohammadi [26], this specific study did not find a significant correlation between EQ and time management overall. However, upon further analysis of the sub-domains of EQ, the study revealed a noteworthy and direct correlation between the level of social awareness of EQ and overall time management and time management mechanics, goals and priorities, as well as perceived time control.

Two cross-sectional studies analysed the relationship between EQ and employee performance. One cross-sectional study distributed surveys amongst leaders and their employees at a hospital in Nigeria to assess leaders’ EQ and employee performance. The study concluded that while the direct impact of leadership style on employees’ performance was not statistically significant, the introduction of a moderation effect revealed that leadership style does moderate the relationship between leaders’ EQ and employees’ performance. This implies that the combination of leadership style and EQ can have a more significant influence on how well employees perform [17]. The relationship between leader EQ and employee performance was examined in the Lebanese healthcare sector [15]. In a cross-sectional study involving 188 employees and 30 managers, it was observed that managers’ EQ had a significantly positive impact on employee performance, with social skills being the most influential variable affecting employee performance. Based on the findings from both of these studies, it can be concluded that there is a positive relationship between a leader’s EQ and employee performance.

### 4.5. Study Limitations and Recommendations

While this review highlights the positive impacts of EQ in healthcare leadership, several limitations must be acknowledged. Firstly, the majority of studies included are cross-sectional, which limits the ability to establish causality between EQ and observed outcomes, such as job satisfaction, emotional exhaustion and team performance. Longitudinal studies would provide stronger evidence for causal relationships. Secondly, there is considerable heterogeneity in the study methodologies and metrics used to assess EQ and the related outcomes, making it challenging to compare results across studies and draw definitive conclusions. This heterogeneity also includes varying definitions of proxy performance metrics, potentially impacting consistency and interpretation.

Additionally, most studies relied on self-reported data, introducing potential biases such as social desirability bias, which may affect the reliability of the reported outcomes. Another limitation is the geographic and cultural scope of the studies, as many were conducted within specific regions or cultural contexts. This narrow scope reduces the generalisability of the findings, given that cultural differences can significantly influence leadership styles, team dynamics and perceptions of EQ.

Finally, this review found limited research exploring the direct impact of EQ on patient satisfaction, a key metric for assessing healthcare outcomes. To address these limitations, future research should incorporate longitudinal studies and broader cultural contexts. Moreover, there is a need for tailored AI training programmes that integrate EQ development according to the specific demands of different healthcare environments. For example, high-stress areas such as intensive care units (ICUs) may require training focused on resilience and stress management, whereas administrative settings could benefit from training that emphasises interpersonal communication and team cohesion. These tailored programmes can support healthcare leaders in applying EQ skills effectively in diverse roles, ultimately contributing to improved organisational and patient outcomes.

## 5. Conclusions

This systematic literature review analysed the impact of EQ in healthcare leaders on satisfaction, emotional exhaustion, job performance and organisational culture. The findings indicate that higher EQ positively influences job satisfaction, reduces burnout and stress, fosters teamwork and enhances job performance. These factors align with the patient-centred care model [27], which is increasingly valued in healthcare. However, no peer-reviewed literature directly assessed the impact of EQ on patient satisfaction.

To address the study limitations, future research should include longitudinal studies to establish causality, as well as cross-cultural studies to broaden the generalisability of the findings. Additionally, specific EQ training programmes tailored to diverse healthcare contexts are recommended to maximise the benefits of emotionally intelligent leadership.

## Figures and Tables

**Table 1:** Inclusion criteria.

Inclusion Criteria
Mention of/focus on emotional intelligence
Participants hold formal leadership role
Mention of/focus on healthcare setting
Mention of/focus on (proxy) performance metrics
Primary research
Peer-reviewed studies

**Table 2:** An overview of study characteristics, aims and main findings.

Authors (Year of Publication)	Title	Population	Country	Aim	Results
Sadasivan, Vasanthi Kumari, Suganthi (2015) [8].	Spirituality, emotional intelligence and work stress-a scrutiny	200 leaders at 20 medical colleges	India	To examine the interassociation between spirituality, emotional intelligence and work stress among leaders in the healthcare sector	Increased emotional intelligence scores correlated significantly with decreased work stress.
Fathi and Kazemeini (2013) [9].	Emotional Intelligence as an Effective Tool in Improving Performance of Leaders	100 managers in the Ministry of Health and Medical Education	Iran	To determine the relationship between emotional intelligence and stress and definition of management performance of headquarters of the Health and Medical Education Ministry through path analysis.	A statistically significant positive relationship was found between EQ and directors’ performance. EQ has a direct and indirect impact (through the reduction in stress) on the performance of directors.
Rinfret, Laplante, Lagacé, Deschamps, Privé (2018) [10].	Impacts of leadership styles in health and social services: A case from Quebec exploring relationships between emotional intelligence and transformational leadership	171 employees working closely with executive directors (EDs)	Quebec	To ascertain the positive influence of the emotional intelligence and transformational leadership (TL) of executive directors during the implementation of reforms in healthcare establishments.	The results show a positive relationship between EQ and TL of EDs in healthcare. This EQ is associated with the perception of justice, which, in turn, is connected to increased commitment and motivation in the workplace. These factors are negatively associated with stress and emotional exhaustion.
Codier, Kamikawa, Kooker (2011) [11].	The Impact of Emotional Intelligence Development on Nurse Managers	15 nurse managers	Hawaii	To explore the impact of a peer coaching intervention on EI abilities of nurse managers.	Total EI score and satisfaction with work–life balance correlated positively.
Erkutlu and Chafra (2012) [12].	The impact of team empowerment on proactivity: The moderating roles of leader’s emotional intelligence and proactive personality	910 certified nurses in 82 teams	Turkey	To investigate the relationship between team empowerment and team proactivity and the moderating roles of a team leader’s EQ and team members’ proactive personality.	Team proactivity is positively influenced by team empowerment, with team leaders’ EQ and team members’ proactive personality playing a significant role in this relationship. Specifically, higher levels of team proactivity are observed when both the team leader’s emotional intelligence and the team members’ proactive personality are high.
Bikmoradi, Abdi, Soltanian, Dmoqadam, Hamdi (2018) [13].	Nurse managers’ emotional intelligence in educational hospitals: A cross-sectional study from the west of Iran	370 nurse managers	Iran	To investigate nurse managers’ EQ in educational hospitals of Hamadan University of Medical Sciences.	There was significant relation between the mean score of nurse managers’ emotional intelligence and gender, age, marital status, number of children, educational level, work experience, managerial work experience, job and life satisfaction.
Majeed and Jamshed (2020) [14].	Nursing turnover intentions: The role of leader emotional intelligence and team culture	344 nurses working in teams in private hospitals	Pakistan	To explore the influence of leader emotional intelligence on the working culture prevailing in teams that ultimately impacts nurses’ intent to leave the job.	The results indicated that leader EQ plays a positive role in suppressing the turnover intentions of the nursing staff. This relationship was found to be strengthened with a conducive team culture.
Vrontis, El Chaarani, El Nemar, Dib (2021) [15].	The relationship between managers’ emotional intelligence and employees’ performance	188 employees and 30 managers	Lebanon	To understand how EQ can improve employee’s performance and to examine the relationship between managers’ EQ and employees’ performance.	The results reveal a positive and significant impact of managers’ EQ on employees’ performance.
Tofighi, Tirgari, Ghomian, Safari, Bazyar, Mohammadi, Malekyan, Safarpour (2022) [16].	Time Management Behaviors and Emotional Intelligence in Head Nurses in Emergency and Intensive Care Units	38 nurse managers of emergency and intensive care units	Iran	To assess the relationship between time management and EI and the level of EI and time management skills in head nurses in emergency and intensive care units.	There was no significant relationship between overall EQ and time management skills. There was a significant correlation between social awareness and overall time management skills.
Oyewunmi, Ojo, Oludayo (2015) [17].	Leaders’ emotional intelligence and employees’ performance: A case in Nigeria’s public healthcare sector	50 leaders and 150 subordinates	Nigeria	To explore the impact of leaders’ emotional intelligence on employees’ performance within Nigeria’s public healthcare sector.	Leadership style has a moderating effect on the relationship between leaders’ emotional intelligence and employees’ performance.
Tyczkowski, Vandenhouten, Reilly, Bansal, Kubsch, Jakkola (2015) [18].	EI and Nursing Leadership Styles among Nurse Managers	77 nurse managers completed all surveys	USA	To determine the level of and relationship between EI and leadership style of nurse managers employed in Wisconsin and Illinois facilities.	Statistically significant positive relationships were noted between EI and transformational leadership and the outcomes of leadership (extra effort, effectiveness and satisfaction).

**Table 3:** JBI Scores for included studies. Red, orange and green represent a high, medium and low risk of bias, respectively.

Study	Critical Appraisal Score
Sadasivan, Vasanthi Kumari, Suganthi (2015) [8].	75%
Fathi and Kazemeini (2013) [9].	62.5%
Rinfret, Laplante, Lagacé, Deschamps, Privé (2018) [10].	75%
Codier, Kamikawa, Kooker (2011) [11].	73%
Erkutlu and Chafra (2012) [12].	100%
Bikmoradi, Abdi, Soltanian, Dmoqadam, Hamdi (2018) [13].	75%
Majeed and Jamshed (2020) [14].	75%
Vrontis, El Chaarani, El Nemar, Dib (2021) [15].	62.5%
Tofighi, Tirgari, Ghomian, Safari, Bazyar, Mohammadi, Malekyan, Safarpour (2022) [16].	62.5%
Oyewunmi, Ojo, Oludayo (2015) [17].	50%
Tyczkowski, Vandenhouten, Reilly, Bansal, Kubsch, Jakkola (2015) [18].	62.5%

## Data Availability

This systematic review is based on data extracted from publicly available studies included in the review. All relevant data are within the paper. No new primary data were generated in the course of this study. For further details on the studies included, please refer to the original publications cited within the manuscript.

## Appendix A. Search Terms and Strategy for Article Retrieval

**Table:**

Keyword	Emotional Intelligence	Performance Indicators	Healthcare	Leadership
Search String	‘emotional intelligence’ OR ‘EI’ or ‘emotional quotient’	‘performance indicator*’ or ‘performance measure*’ or ‘performance metric*’ or ‘burnout’ or ‘satisfaction’ or ‘job satisfaction’ or ‘patient satisfaction’ or ‘stress’ or ‘occupational stress’ or ‘turnover’	‘healthcare’ or ‘national health service’ or ‘nhs’ or ‘healthcare professional’ or ‘doctor*’ or ‘nurs*’ or ‘midwi*’ or ‘therapist*’ or ‘surg*’ or ‘pharmac*’ or ‘*medic*’	‘leader*’ or ‘team leader*’ or ‘command*’ or ‘mentor*’ or ‘Consultant*’ or ‘influenc*’ or ‘clinical director*’ or ‘executive director*’ or ‘matron’ or ‘medical director’ or ‘supervis*’

Truncation character (*) used to search for multiple matches of word.

Each search term combined with AND.

Limits Adopted:

- English Language
- (January 2009–May 2023)

Inclusion Criteria:

Mention of/focus on emotional intelligence
Participants hold formal leadership role
Mention of/focus on healthcare setting
Mention of/focus on (proxy) performance metrics
Primary research
Peer-reviewed studies
